# The next wave: key adaptations to operational workflows of National Screening Centre (Singapore) and the emergency department during the COVID-19 pandemic

**DOI:** 10.1186/s12245-021-00337-w

**Published:** 2021-02-24

**Authors:** Charmaine Malenab Manauis, Marvin Loh, Amanda Hui Jun Lim, James Kwan, Han Jie Teo, David Kuan Peng Teng, Shawn Sushilan Vasoo, Yee Sin Leo, Hou Ang

**Affiliations:** 1 Emergency Department, Tan Tock Seng Hospital, Singapore, Singapore; 2 National Centre for Infectious Diseases, Singapore, Singapore; 3 Department of Medicine, Yong Loo Lin School of Medicine, National University of Singapore, Singapore, Singapore; 4 Lee Kong Chian School of Medicine, Singapore, Singapore

## Introduction

In the initial phases of the COVID-19 pandemic, Singapore received international recognition for her outbreak response and was regarded as the “gold standard” by international researchers [1]. The situation has evolved markedly since. By April 2020, rapid transmission amongst the migrant workers residing in dormitories led to a sudden surge in the number of cases. This vulnerable sector presented a fresh set of challenges to our healthcare system.

Similar to Singapore, migrant workers form a significant proportion of the workforce for many countries across the world, including those in North America, Europe, and parts of Asia [2]. Other vulnerable sectors include highly populated, closely packed urban residential areas and incarcerated populations such as prison facilities or even refugees living in overcrowded detention camps [3, 4]. A previous review of infectious diseases amongst migrant workers in Singapore had highlighted the significantly higher risk of transmission of communicable diseases within this vulnerable group, underlining the unique challenge of curbing transmission in crowded living conditions [5].

This paper aims to describe the key adaptations in workflow [6] in the National Centre for Infectious Diseases (NCID) Screening Centre (SC) and Tan Tock Seng Hospital (TTSH) Emergency Department (ED), respectively, in response to the evolving challenges encountered in the COVID-19 pandemic.

### The first wave (January to March 2020)

Incorporating her previous experiences combating the 2003 severe acute respiratory syndrome coronavirus (SARS-CoV) outbreak [7], Singapore adopted a rigorous containment strategy involving aggressive swab testing, rapid contact tracing and quarantine of positive cases and close contacts.

One of the important keys to Singapore’s containment efforts has been the SC which has been fully operational since 31 January 2020 and housed in NCID, a purpose-built facility which opened in September 2019, dedicated to the containment of infectious disease outbreaks [8]. NCID is located adjacent to its partnering hospital, TTSH. Emergency physicians from TTSH, in close collaboration with their colleagues from the Infectious Diseases Department, helm operations at both the SC and the adjacent Emergency Department. To sustain round the clock operations, a “Whole-of-Hospital” approach was adopted with and assistance of doctors from the other departments including General Surgery, Orthopedic Surgery, Hand Surgery, Neurosurgery, Radiology, Pathology, Ophthalmology, Otorhinolaryngology and Urology who took up roles as medical officers in the SC. Nursing and administrative staff were also recruited from the different departments and clinics.

The initial efficacy of Singapore’s containment strategies has been demonstrated in a recent study which analysed the first 100 positive cases of COVID-19 in Singapore (up to 29 February 2020) [9]. The 7-day moving average of the interval from symptom onset to isolation during the 1-month study period declined significantly for both imported and local cases, from 9.0 and 18.0 days to 0.9 and 3.1 days.

### The second wave (April 2020 to present)

**Fig. 1:**
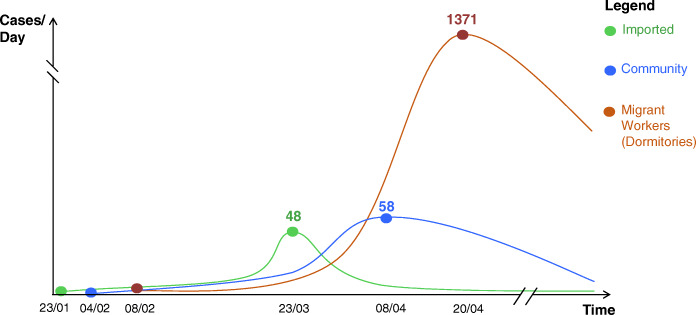
Number of confirmed COVID-19 cases per day (not to scale, information updated as of 31 May 2020)

By April 2020, the situation had evolved in 3 main ways (see Fig. 1). Firstly, the number of imported cases started to decline from its peak of 48 cases. Secondly, there was a gradual climb in the number of community cases, with a peak of 58 cases on 8 April 2020. This included an average of more than 10 unlinked cases a day. Finally, there was a sharp rise in positive cases amongst the migrant worker population with a peak of 1371 cases on 20 April 2020.

## Adaptations to workflow in NCID SC

### Background

The SC is housed in NCID, which is a 330-bed facility with general and ICU wards with negative-pressure isolation rooms [8]. NCID also houses the National Public Health Laboratory equipped with a biosafety-level 3 containment facility. The SC is used for small-, medium- and large-scale outbreaks. It is equipped with negative-pressure isolation rooms, procedure rooms, radiology facilities and pharmacies. Individual consultation rooms are available for non-ambulatory patients. The SC also has resuscitation rooms staffed round the clock by specialist Emergency Physicians. The air in the SC undergoes a change at a rate of 12 per hour, is High-Efficiency Particulate Air (HEPA) [10] filtered and undergoes UV treatment. The staff rest area utilises a positive-pressure air regime whereas the resuscitation room and procedure rooms utilise a negative-pressure air regime as some of our patients may require procedures at risk of aerosolisation such as intubations, suctioning and nebulisations.

### Active decantment of cases

The size of Singapore’s migrant worker population is estimated to be approximately 1.4 million (out of a total population of 5.6 million), according to a recent census conducted in 2019 [11]. This includes 323,000 migrant workers who reside in dormitories and are mainly involved in the country’s construction industry. The typical room in a purpose-built dormitory has a floor area of approximately 50 m^2^ and can house up to 10 workers [12], making physical distancing a significant challenge.

During this COVID-19 pandemic, transmission amongst the migrant workers residing in dormitories was particularly rapid. The number of positive cases amongst the migrant worker population ranges from 300 to more than 1000 cases daily. Between April and May 2020, there were more than 30,000 new cases diagnosed [13], exerting significant stress on our healthcare capacity. At NCID SC and TTSH ED, the proportion of migrant workers screened has more than doubled from 30.9% in March to 87.0% in April 2020 (refer to Table 1).

**Table 1:** Demographic information of patients presenting for screening from 1 Feb to 31 May 2020

*N*= 30,965 (100%)				
	Feb	Mar	Apr	May
Total attendance – no.	4926	9951	8929	7159
Mean Age, Years (Median)	38 (35)	39 (35)	39 (35)	40 (35)
Male sex (%)	50.0%	51.2%	76.6%	89.2%
Residence status (%)				
Citizens or Permanent Residents	63.3%	69.1%	13.0%	17.4%
Migrant workers	36.7%	30.9%	87.0%	82.6%
Disposition (%)				
Decanted to CCF	Not applicable		24.1%	37.3%
Admitted	15.5%	6.3%	21.6%	13.7%
Discharged	76.1%	77.1%	57.6%	43.0%

In contrast to other health systems from countries such as the USA and Switzerland where positive cases with mild symptoms are discharged back to their own homes and instructed to practise strict self-isolation [14, 15], Singapore’s approach is to admit migrant COVID-19-positive patients to the hospitals or dedicated isolation facilities. Previous studies have demonstrated successful containment of outbreaks via isolation measures with reduction in the reproductive number, *R*_0_, a measure for transmission [16]. Admission at a hospital or dedicated isolation facility allows for assessment of illness severity at the point of presentation, utilising radiological investigations such as chest radiography as well as laboratory tests such as the C-reactive protein and lactate dehydrogenase to better prognosticate and optimise siting of care.

Our initial response was to increase the bed capacity to house the large number of new positive cases. As part of contingency arrangements, NCID was built with a flexible design that allows rapid ramping up of bed capacity from 330 to 586 beds. Despite an expanded capacity, the sheer volume of new positive cases still resulted in intense bed pressure, with the daily bed occupancy rate averaging more than 90%. This called for other alternative solutions which were promptly adopted.

**Fig. 2:**
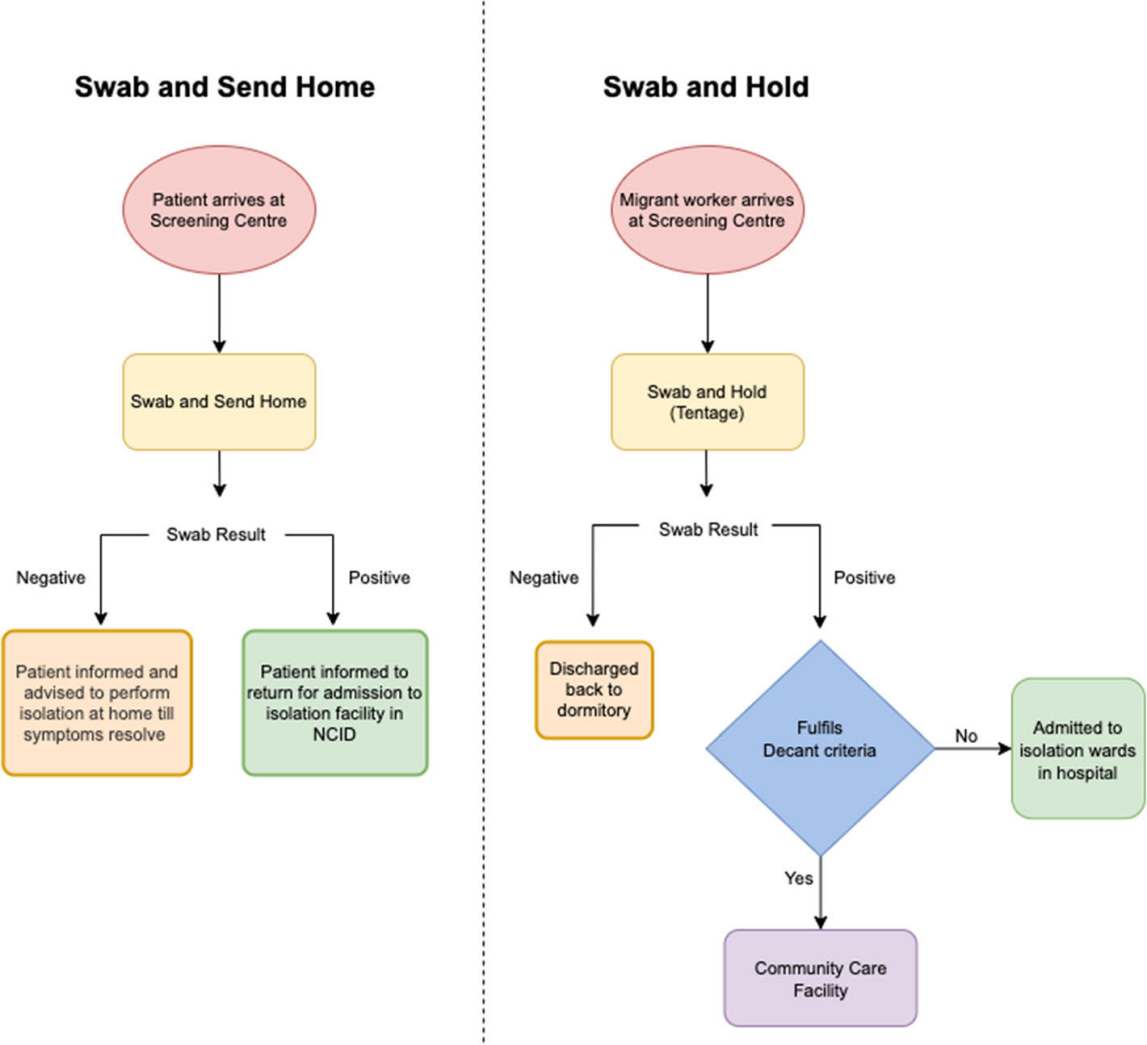
New disposition workflow (swab and hold) for migrant workers living in dormitories

Firstly, we pursued a strategy involving active decantment of cases (refer to Fig. 2). This included patients already hospitalised in NCID, as well as patients who tested positive at the SC. Data published from multiple studies with large patient cohorts [17, 18] have consistently demonstrated that the risk of medically adverse outcomes is significantly lower amongst patients from the younger age groups and those with no chronic comorbidities. Our decantment criteria therefore include age < 45 years, absence of other significant comorbidities and a normal body mass index.

The National Early Warning Score (NEWS) was also utilised to further risk-stratify a subset of patients to determine eligibility for transfer [19, 20]. These positive cases are decanted to community care facilities (CCFs) that have their own medical teams providing on-site medical coverage and surveillance round the clock. Patients who experience worsening of symptoms or clinical status are promptly identified. Initial assessment is performed and patients are referred back to the SC or other hospitals should a higher level of care be deemed necessary. As of end April 2020, the combined bed capacity at the community care facilities was at 10,000, which was subsequently doubled to 20,000 by end June 2020 [21]. These CCFs provide an essential channel for decantment to facilitate the throughput of our SC and alleviate the bed pressure at NCID.

### Swab and hold strategy

Previously, patients were discharged back home while awaiting their swab test results and were instructed to practise strict self-isolation. Positive cases were conveyed back immediately to NCID via designated ambulances. During the second wave which involved mainly migrant worker population, the high prevalence rate (between 8 and 9%) of positive cases amongst the migrant workers residing in dormitories, coupled with the practical challenges in effective physical distancing due to crowded living conditions, meant that we had to revise this strategy. Since April 2020 (refer to Fig. 2), migrant workers residing in dormitories have been held at a designated tentage (within the SC) while awaiting their swab test results and only those who tested negative are discharged. This is to help stem the transmission within these dormitories.

### Clinical evaluation of positive cases

Since April 2020, the SC has been receiving a larger proportion of positive cases as mass testing capability was significantly scaled up in the dormitories and the community. This also includes patients who were previously decanted to community care facilities or community recovery facilities. These patients are referred back to our SC for further evaluation after initial assessment by the on-site medical team. To minimise criss-crossing of patient flows and nosocomial transmission to other patients, a special zone was designated within the SC to hold these positive cases.

## Adaptations to workflow in TTSH ED

### Background

TTSH is the second largest adult tertiary care hospital in Singapore with a bed capacity of 1500. Its ED provides round the clock emergency services and receives close to 450 attendances each day. The ED Infectious Diseases workgroup collaborates closely with the TTSH Department of Clinical Epidemiology and the Department of Infectious Diseases which performs horizon scanning and surveillance of local clusters and global infectious disease threats on a daily basis.

### ED forward triage

**Fig. 3:**
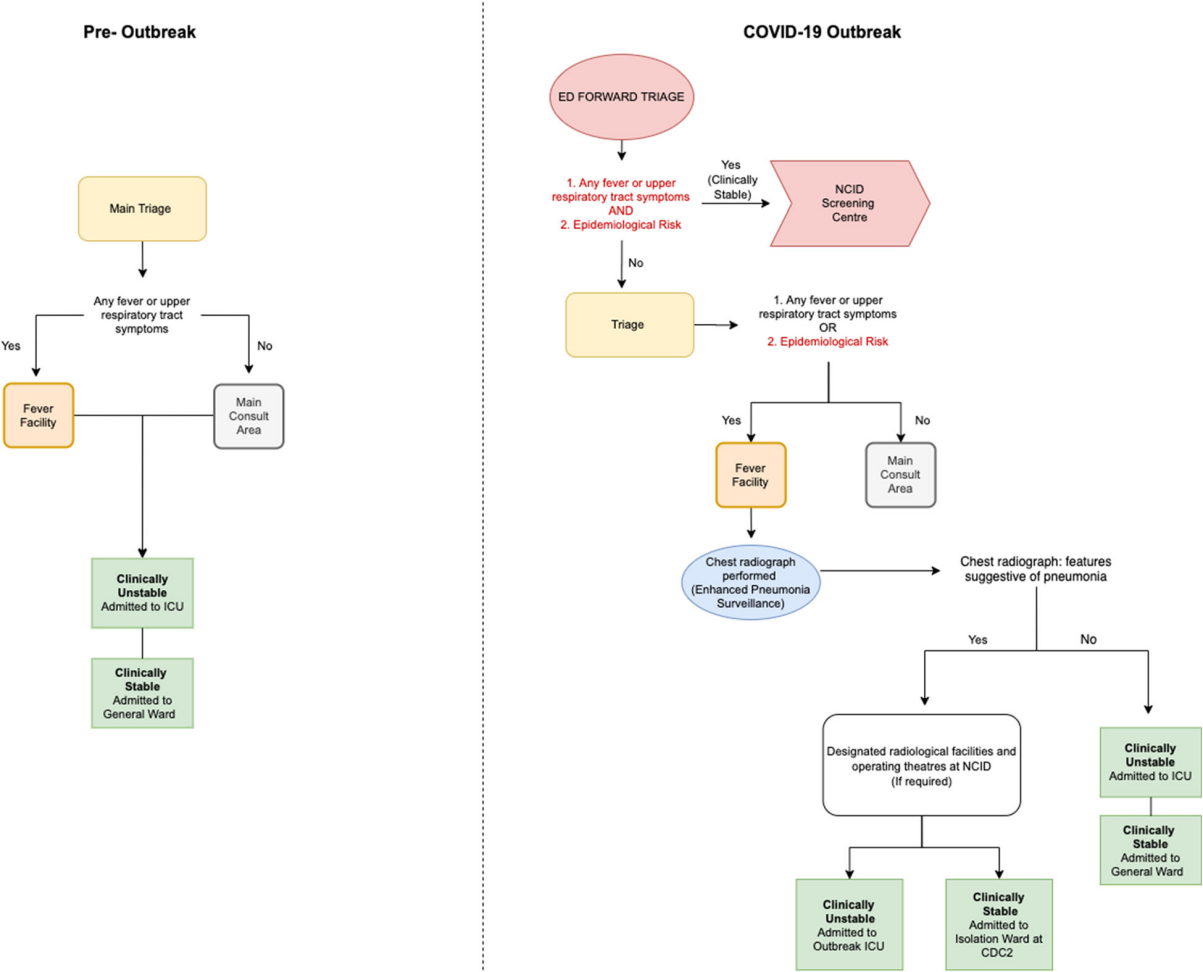
Adaptations to workflow in Emergency Department, Tan Tock Seng Hospital

At the forefront of new emerging infectious diseases, the ED was quick to redesign current workflows to cope with the evolving pandemic. A forward triage point is set up at TTSH ED (refer to Fig. 3) to divert stable higher risk cases to NCID SC to segregate high-risk cases away from TTSH. This includes patients who have respiratory symptoms and epidemiological risk factors such as recent travel, close contact with positive cases or access to high-risk hotspots or clusters.

### Tightening of triage criteria

Previously, patients with any symptoms of fever and/or respiratory tract symptoms were triaged to the fever facility. Given the high prevalence of positive cases amongst the migrant workers, as well as emerging data of asymptomatic cases [22], the triage criteria were further tightened. Migrant workers are now diverted to the fever facility even if they are asymptomatic. As a safety measure, all patients and visitors at the ED are instructed to wear masks and all staff working in the fever facility are required to wear full personal protective equipment (PPE). This consists of a hair cap, goggles, N95 mask, disposable gowns with knitted cuffs and gloves [23]. To reduce the risk of nosocomial spread, bed trolleys and patient chairs are spaced 2 m apart. This is in line with local and international guidelines on droplet precautions [24–26].

### Enhanced pneumonia surveillance (EPS) and Intensive Care Unit Headquarters (ICU HQ)

In addition to the aforementioned measures of a forward triage point as well as tightening of triage criteria, a third layer of protective strategy is that of enhanced pneumonia surveillance. This is in response to the rise in the number of unlinked positive cases, suggestive of some level of silent transmission within the community. The EPS is targeted at patients who have radiological findings suggestive of pneumonia, even when they have mild or even no respiratory symptoms. These patients are sent to a separate radiological facility (refer to Fig. 3) to undergo their radiological investigations. They are subsequently admitted to isolation rooms in a separate location at the adjacent Communicable Diseases Centre 2 (CDC 2). The ED also works closely with the ICU HQ in situations whereby patients require admission to the ICUs. Patients with respiratory symptoms, epidemiological risk factors or radiological findings suggestive of pneumonia are admitted to a designated outbreak ICU. This measure serves to protect other critically ill patients in the ICU. As demonstrated in several studies [27, 28], patients with underlying medical conditions tend to have a significantly higher mortality and morbidity rate. The EPS also protects our downstream colleagues working in the radiological facilities and wards. The ICU HQ is set up to provide operational oversight over the management of critical but limited ICU bed resources, especially in the context where other health systems overseas have had their intensive care resources overwhelmed [29], leading to a catastrophic spike in patient mortality and morbidity as seen in countries such as Italy and China [30, 31].

### Visitation policy

To safeguard the safety of the patients in the ED, especially those who are frail with multiple comorbidities, a no-visitor policy was adopted by the ED. The exception includes patients who are critically ill—in this instance, only 1 designated visitor is allowed, and this visitor has to register with his or her personal particulars for contact tracing purposes. Temperature taking is performed and a screening questionnaire is administered to ensure that the visitor is not symptomatic or has any epidemiological risk factors.

### Resource management

**Fig. 4:**
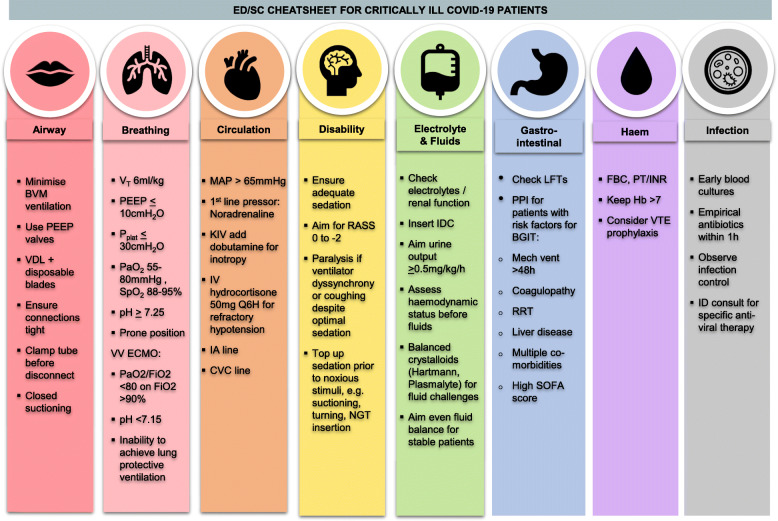
Sample material produced by TTSH ED critical care workgroup

Due to major disruptions in global supply chains [32], medical supplies including commonly used medications in the resuscitation room and ICUs have been in short supply. Our critical care workgroup was tasked with providing guidance to our ED staff on the use of alternative sedative agents so as to conserve our limited supply of propofol and fentanyl. The workgroup prepared refresher materials on the appropriate indications and significant adverse effects of the various medications. In addition, they provided timely updates with regard to the management of critically ill patients with COVID-19 (refer to Fig. 4), with special focus on the unique features of the disease. This includes airway practices such as clamping of the endotracheal tube when disconnecting from the ventilator (to prevent alveolar derecruitment [33] and as an infection control measure) as well as recommendations on key ventilator strategies [34].

## Discussion

Our migrant worker population, particularly those living in dormitories, represent a vulnerable sector for Singapore in this pandemic. Rapid transmission within a vulnerable sector can precipitate a sudden surge in the number of cases as seen in countries such as India, Malaysia, Kuwait, Bahrain and the U.A.E [35].. As observed in multiple health systems across the world, without a comprehensive set of contingency strategies, these surges in patient load can quickly overwhelm healthcare capacity, leading to adverse patient outcomes and spikes in mortality rates. Maintaining a smooth throughput, especially at the frontline, is hence a challenging yet critical task.

In our experience, the aforementioned strategies as summarised in Table 2 have been indispensable in adapting to the evolving COVID-19 situation. During the second wave, a significant proportion of our migrant workers belongs to the younger age group, with a mean age (years) of 39 (refer to Table 1), making an active decantment strategy to community care facilities amenable. Between the months of April and May 2020, 24.1% and 37.3% of total attendances were successfully decanted to CCFs. Active decantment has helped to reduced admission rates by 33.1%, thereby reducing the strain on hospital beds and resources. Only a small proportion of patients (3.6%) in the CCF required a transfer back to a general hospital for further medical evaluation [36]. Singapore’s overall case fatality rate continues to remain low at 0.05% by end of May 2020, the lowest in the world [37].

**Table 2:** Strategies employed during the second wave of COVID-19 infections

	New Trends	Challenges	Strategies
**Patient demographic**	• Surge in number of COVID-19 cases among foreign workers	• Inadequate physical distancing in crowded dormitories	• Purpose-built community isolation facilities to relieve access block in hospitals • Swab and hold - patients are only discharged after a negative swab test result
**Disease presentation**	• Asymptomatic carriers or patients with mild symptoms	• Reliable identification of high risk patients using traditional triage tools	• Tightened triage criteria • Enhanced pneumonia surveillance
**Healthcare resources**	• Significant surge in patient admissions • Disruption to global supply chains	• Shortage in medical supplies, e.g. commonly used drugs in resuscitation room or ICUs	• Guidance provided by ED critical care workgroup on alternative options

## Conclusion

The evolving pandemic will continue to pose significant challenges to healthcare systems across the world. Emergency departments serve a crucial role at the forefront of outbreak responses, providing early triage and acute management. Our emergency response focuses on space, manpower and triage systems. We hope these strategies can be incorporated by other countries in their outbreak response, especially those who encounter similar challenges from their respective vulnerable sectors and communities.

## Data Availability

All data generated or analysed during this study are included in this published article.
